# The effect of food insecurity on health status of adolescents in Ethiopia: longitudinal study

**DOI:** 10.1186/s12889-017-4406-5

**Published:** 2017-05-18

**Authors:** Mulusew G. Jebena, David Lindstrom, Carl Lachat, Tefera Belachew, Patrick Kolsteren

**Affiliations:** 1 Department of Population and Family Health, Jimma University, P.O. Box 378, Jimma, Ethiopia; 2 Department of Food Safety and Food Quality, Ghent University, Coupure Links 653, 9000 Ghent, Belgium; 3 Department of Sociology, Brown University, Box 1916, Maxcy Hall, Providence, RI 02912 USA

**Keywords:** Food insecurity, Social determinants, nutrition, Health status, Adolescents, Southwest Ethiopia

## Abstract

**Background:**

The effect of food insecurity on health and wellbeing of a population has been the subject of much research. Yet, limited research has investigated its effect on adolescents’ health and wellbeing in Ethiopia.

**Method:**

We used data from the Jimma Longitudinal Family Survey of Youth which began tracking a cohort of adolescents in 2005 to examine the social, behavioral and economic determinants of their health and well-being. A total of 1,919 sample were included in the main analyses. All youths provided data related to their food insecurity experiences and their health status. A mixed effect logistic regression using random intercept and trend model was used to examine the relationship between food insecurity and their health status. Fixed effects estimates were also computed to check the parsimoniousness of the random intercept and trend model.

**Results:**

The results indicated that the mean (±SD) age of adolescents was 18.6(±1.4). Nine hundred twenty three (48.1%) of them were female. The magnitude of self-rated health status was relatively unstable ranging from 18.9%, 34.7% to 37.3% in each round. Similarly, 20.4%, 48.4% and 20.6% of adolescents were food insecure during each consecutive round of the survey respectively. Exposure to food insecurity is strongly associated with self-rated health status (β = 0.28, *P* < 0.001) and poor self-rated health was also more pronounced for some time (β =2.11, *P* < 0.001) and decline after a turning point (β = −0.38, *P* < 0.001).

**Conclusions:**

These findings imply that any social, nutrition and public health interventions designed to improve adolescent health should consider underlying social determinants of health such as food insecurity.

**Electronic supplementary material:**

The online version of this article (doi:10.1186/s12889-017-4406-5) contains supplementary material, which is available to authorized users.

## Background

Life course theories indicated that exposures to health risks such as food insecurity could have a profound effect on health outcomes, on the onset of diseases and its progressions in later life [1–3]. Food insecurity is a pressing social and public health problems of low and middle income countries. It is associated with poorer health outcome [4], self-rated health status [5], depression and anxiety [6–8], reduced micronutrient intake, fruit and vegetable consumptions [9], obesity and overweight [10], poor child growth [11] and birth defects [12].

Poor self-rated health status (SRH) is also one of the health outcomes associated with food insecurity [5]. Self-rated health (SRH) reflects both subjective and objective measures of overall health status [13]. It is a social, biological and psychological dimension of health and could predicts health inequities, morbidities, mortalities and health care utilizations. As such, SRH has been used as a measure of social inequities in health [14–17].

A growing body of evidence have explored the multitude determinants of SRH. These include, but not limited to low socioeconomic positions and household food insecurity [5, 15, 18–21]. Similarly, the effect of food insecurity on health and well-being has been the subject of much research. However, very little information exists on SRH status of adolescents and limited research has attempted to document the effect of food insecurity status on adolescent health and well-being in the context of low and middle income countries [20, 22–25]. With few exceptions, research in Ethiopia has also focused more on exploring determinants of food insecurity and few studies showed the association of food insecurity on adolescent morbidities and poor health outcomes [20, 26–28]. Nevertheless, there were limitations to existing literatures (1). Many studies were cross-sectional in nature and do not capture information on the effect of long term exposures to food insecurity on their health outcomes. To the best of authors’ knowledge, no research in low- and middle -income countries in general and Ethiopia in particular has evaluated the relationships between exposures of food insecurity and adolescent health and wellbeing using longitudinal data (2). Even if the research is found, the responses of both health outcome and food insecurity were depend on the head of the households and household level food insecurity status rather than adolescents’ own experiences. (3). Most of these studies highly focused on adult, women and children by ignoring its potential health consequences on adolescents’ self-rated health. This is unfortunate as perceptions about one’s own health status and the trajectory effect of food insecurity formed during adolescence could persist into adulthood and may have far reaching implications on morbidity, mortality and health-care utilization [13, 18, 26, 29]. Thus, we assume the underlying relationships between food insecurity and health status on other populations such as children and adults could also holds true for adolescents**.** Therefore, the aim of this study was to examine the effect of food insecurity on self-rated health status of Ethiopian adolescents using repeated measurements after controlling for other factors. Exploring such evidence would generates an additional information helpful to design effective social, nutrition and public health policy for Ethiopian adolescents from life course perspectives [3, 30, 31].

## Methods

### Study design, setting and sampling

The Jimma Longitudinal Family Survey of Youth (JLFSY) began tracking a cohort of adolescents since 2005 to examine the social, behavioral and economic determinants of adolescent their health and well-being. The study was carried out in Southwest Ethiopia encompassing 18 study sites spread over four Woreda (districts) from rural, semi-urban, and urban communities to represent a range of ecological and development contexts. A two-stage sampling plan was used to select eligible adolescents for the study. On the first stage, households were randomly sampled from within each study site with the sample size in each site is determined by the relative proportion of the study population in the site and the overall target sample size. At this stage a total of 3700 households having at least 1 male or 1 female adolescent were identified. On the second stage, one adolescent aged 13 to 17 years was randomly selected from each household using a Kish Table. The detail sampling procedures were described earlier [20, 26, 32, 33]. A total of 2084 (100%), 1919 (92.1%), 1465 (70.3%) of youths were interviewed during the first round (mid 2005/06), second round (mid 2007/08) and third round (2009/10) respectively. We used a structured questionnaire developed in English language and translated back to local languages (Amharic and Afan Oromo) to collect the data. The questionnaire was used by previously published works [20, 28, 34]. The head of the household responded to the household questionnaire and adolescents’ questionnaire focused on issues related to personal experiences of food insecurity, self-rated health, education, nutritional status and anthropometric measurements. The interview was conducted in a private way by an interviewer of the same sex (i.e. boys were interviewed by male interviewers and girls were interviewed by female interviewers). The same interviewers remained with the same households and adolescent respondents throughout the study periods.

### Measurements

#### Health status

Adolescent health status was assessed using a one item question of self-reported measures of health where an individual was asked “In general, how would you rate your health today?” to rate their perceived SRH status. This item is considered as valid and reliable measure of current health status [15, 18, 35–37]. Possible responses to the first question were very good “0”, good “1”, moderate/fair “2“, and poor/bad “3“. The answers were dichotomized into “good=0” by combining “good” and “very good” and coding the rest as “poor = 1.

#### Adolescent food insecurity status

Adolescent food insecurity status was measured using a 5-items point interval scale. Briefly, respondents were told to think about their own experiences in the last three months if they had experienced the following conditions.

(1). How many days did you worry that you would run out of food or not have enough money to buy food?

(2). How many days have you had to reduce the number of meals eaten in a day, because of shortages of food or money?

(3). How many days have you had to reduce the size of meals eaten in a day, because of shortages of food or money?

(4). How many days have you had to spend the whole day without eating, because of shortages of food or money? And.

(5). How many days have you had to ask for food or money to buy food?

The responses to the above questions included (1). “Never”, (2). “1–7 days”, (3).8–21 days” and (4). “> 21 days”*.* Food insecurity experience reports were coded as “1″ and never responses were coded as “0″, and the responses were summed to produce an index of adolescent food insecurity. We dichotomized the score to represent food secure (summed score of zero) and food insecure (summed score greater or equal to one) adolescents.

#### Household food insecurity status

We measured household food insecurity using a questionnaire adapted from previous studies and using the similar items in the study area [20, 26, 29, 33, 38–40]. To measure household level food insecurity status, a series of six questions covered whether (1) the respondent worried about running out of food; (2) the household ran out of food; (3) the variety of food for children was reduced; (4) the children did not have enough to eat; (5) the respondent or another adult did not eat enough; and (6) the respondent ever felt hungry but did not eat. The “yes” responses were coded as “1” and the “No” responses were coded as “0”, and the responses were summed to produce an index of household food insecurity. Finally, the index was categorized in to food secure and food insecure household. Chronic household food insecurity was defined as the proportion of households who responded affirmatively to at least one of the food security items in all rounds of the survey.

#### Dietary diversity

A food-frequency questionnaire containing 30-food items was used to measure dietary diversity [33, 41–44]. Participants were asked to report the frequency of consumption of each food using the past three months as a reference. Adolescents were coded as a “consumer” of a food item if he/she had consumed the food item at least once per week. A principal component analysis was used to generate a dietary diversity score. The detailed procedures were described in the previous published work from similar data sets [26, 45].

#### Body mass index

Height was measured using standard procedures to the nearest 0.1 cm using a stadiometer (SECA, Hannover, Germany), and weight was measured to the nearest 0.1 kg using digital scales (SECA). BMI was computed by dividing weights for height squared. BMI was adjusted for age and sex and for those aged greater than 19 years old BMI was computed based on WHO classification [46]. We then categorized groups in to underweight (BMI < 18.5 kg.m^−2^), normal (18.5 kg.m^−2^ < =BMI < 25 kg.m^−2^) overweight (> = 25 kg.m^−2^). BMI was used as a categorical variable in the model.

#### Physical health

To measure the perceived wellbeing of adolescents, experience of physical health problems such as headache, stomachache, difficulty of breathing, a sore throat or a cough, feeling sick, aches, pains, or soreness in muscles or joints, diarrhea, ulcer and injury were asked. Response to these morbidity questions was binary and recoded 1 for “Yes” and 0 for “No” and if yes the time of illness were asked “When was the last time you were sick with any illness?” and responses to this question were re-coded into two categories: within the last month or more than one month ago.

#### Other covariates

Other potential covariates were such as age, sex, educational status, female-headed household, parental education, residence; risk factors, physical health, social network and support were identified a priori. Household wealth index was computed based on household ownership of 21 durable items. The index ranged from 0 (not owning any of the items) to 21 (owning all of the items). A principal component analysis method to generate scores of wealth index by differentially weighing each item and this was treated as continuous variable showing the higher scores represented the higher wealth status. Internal consistency of items was adequate (Cronbach’s alpha =0.83).

#### Data management and analyses

Data were analyzed using Stata software (StataCorp, L.P, Stata 13). All survey data were doubled-entered, checked for missing values, outliers, normality assumptions before data analysis. All adolescents who have at least two data points were considered for the main analyses. To assess changes in SRH, physical health and food security status overtime, cross tabulations, test re test reliability of SRH was checked and the results were summarized with the Kappa and intra-class correlation coefficients. The means and standard deviations for SRH and physical and food insecurity status obtained over time were also computed using one-way ANOVA tests.

A mixed effect logistic regression (i.e. random intercept and trend model) analysis was used to account for the longitudinal data structures using the following equation.

1 $$ {SRH}_{ij}={\beta}_0+{\beta}_1{t}_{ij}+{\upsilon}_{0 i}+{\upsilon}_{1 i}{t}_{ij}+{\varepsilon}_{ij} $$

Where i = individuals (1... N), and j = indexes the three rounds of data. *β*_0_ is overall population intercept, *β*_1_ is overall population slope, *υ*_0*i*_ is the intercept deviation for subject i, and *β*_1_ is the slope deviation for subject I; *ε*_*it*_ is an independent error term assumed to be distributed normally. SRH was time varying dependent variables (Time 1 to Time 3) and the correlation structure was assumed exchangeable.

In this, analysis, the effect of food insecurity was estimated using a random intercept and trend model. In addition, unobserved time invariant effects of covariates were accounted for the fixed-effects model. A hierarchical regression model was used where a block of variables were entered sequentially. First, adjusting for time, the sole effect of food insecurity on self-rated health was regressed (**Model I**). In the second model (**Model II**), we included the socio demographic and economic variables (wealth index, gender, parental education, educational status of adolescents, age, residence and religiosity index). Finally, we added variables considered as risk factors and social network such as exposures to chronic household food insecurity, physical illness in the last one month, practicing multiple risk behaviors, diet diversity score BMI and social network (**Model III**). To check the parsimoniousness of the random intercepts model, we did a separate fixed effect estimates accounting for only time variants variables**.** Later, the interaction between food insecurity and period (time) was checked. In addition, variance inflation factors were checked at every step to test for the multicollinearity between explanatory variables. See the collinearity diagnostics of the final model (Additional file 1: Table S1). Robustness of the results was checked by the log likelihood ratio test. The criterion for statistical significance was set at 0.05 and all tests were two-sided. To examine possible bias of adolescents lost to follow up on the findings, we conducted separate analyses and found there was no significant observation difference in their socio economic status, gender differences, food security status and outcome variable, SRH status.

## Results

### Descriptive statistics

A total of 1919 (92.1% of the total sample) adolescents were included in the main analyses. The mean (SD) age of the respondents at round three was 18.6(1.4) Years. The majority, 1171(61.0%) of the respondents were Muslim, 996(51.9%) of them were male and 832 (43.4%) of their parents did not attend any formal education (Table 1).

**Table 1:** Baseline characteristics of study participants, Jimma Longitudinal Family Survey of Youth, Southwest Ethiopia (*n* = 1919)

Characteristics	Number	Percentage
Variables		
Religion		
Muslim	1171	61.0
Orthodox	633	33.0
Others^a^	115	6.0
Sex		
Male	996	51.9
Female	923	48.1
Age, mean (SD)	18.9 ± 1.4	
Educational status		
No schooling	519	27.1
Primary	760	39.6
Secondary and above	640	33.3
Chronic food insecurity (Adolescents)	268	14.0
Body mass index (*n* = 1800)		
Underweight	655	36.4
Normal	1114	61.9
Overweight	31	1.7
Parent education (*n* = 1918)		
Illiterate	832	43.4
Primary	395	20.6
Secondary	691	36.0
Household food insecurity Round1	1172	61.1
Household food insecurity Round2	1660	86.5
Chronic food insecurity (Households)	1087	56.6
Household wealth status (*n* = 1894)		
Low	632	33.4
Average	636	33.6
High	626	33.0
Female headed households	342	17.8
Residence		
Urban	672	35.0
Semi-urban	551	28.7
Rural	696	36.3

*BMI* Body Mass Index

^a^Others include Protestants, Catholics, and Adventists

### Magnitude of food insecurity and self-rated health

**Fig. 1:**
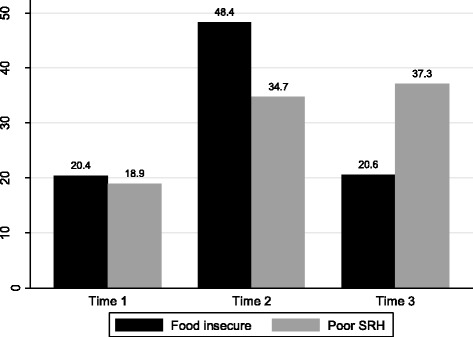
Magnitude of food insecurity status and poor self-rated health among adolescents across the three rounds of the survey, Jimma Longitudinal Family Survey of Youth, Southwest Ethiopia

In this study, 20.4%, 48.4% and 20.6% of adolescents experienced food insecurity and 18.9%, 34.7% and 37.3% of them reported poor SRH over the three rounds of the survey, respectively (Fig 1).

### Stability of self-rated health, physical health & food security status

Table 2 presents Kappa and Rho coefficients. SRH and illness during the last one month were slightly less stable over repeated observations when compared with the other measures. Almost all of variables including SRH and illness in the last one-month have the lowest Kappa and ICC. In other words, adolescents changed their self-rated health more frequently than other measures of physical health and food security status. Inconsistency between SRH and physical health over time was depicted that SRH becoming worse over time while their physical health status was getting better (Table 2).

**Table 2:** Stability of self-rated health, physical health, multiple risk behaviors and food insecurity status, Jimma Longitudinal Family Survey of Youth, Southwest Ethiopia

Variables	Time1^a^	Time2^a^	Time3^a^	Kappa^b^	ICC^c^
Poor SRH	0.19(.39)	0.35(.47)	0.37(.48)	0.12	0.14
Physical Health	3.40(1.80)	1.54(2.02)	0.99(1.69)	0.14	0.10
Multiple risk behaviors	0.46(0.65)	0 .45(.62)	0.55(.85)	0.07	0.13
Feeling tired	0.38(.48)	0.21(.40)	0.19(.39)	0.06	0.13
Had a difficulty/lack energy	0.32(.47)	0.16(.37)	0.14(.35)	0.12	0.13
Illness in the last 1 month	0 .26(.44)	0.14(.35)	0.12(.32)	0.03	0.04
Food insecure	0.20(.40)	0.48(.49)	0.20(.40)	0.10	0.13

*SRH* Self-Rated Health, *ICC* Intra cluster correlation

^a^cell entries depicts means and standard deviations in parentheses

^b^Kappa coefficient for each measure

^c^Intra class correlation coefficient (Rho) for each measure.

### Food insecurity and self-rated health

Table 3 presents the coefficients of random intercept and trend model regressing self-rated health on food insecurity, socio economic positions, risk factors, exposure to physical health (illness) and BMI. Repeated SRH score (Level-1) was nested within individuals (Level-2) and intra cluster coefficients (ICC) showed that 18.9% of the total variance in SRH score occurred at an individual level. In the first model, controlling for time (as a linear and quadratic function), the average food insecure adolescents were 35.7% more likely to report poorer SRH over the survey rounds than their counter parts. But, the quadratic term in this model showed, trends of poor self-rated was higher for some time and started to decline after turning point (β = −0.33). Model II adds those variables assumed to reflect the socio economic positions of the adolescents. Accordingly, this model shows the effect of food insecurity prevails to be associated with adolescents SRH. Food insecure adolescents were more likely to report poor SRH than their counterparts (β =0.33, *p* < 0.001) but the effect of food insecurity decreases by 10. 8% (0.322–0.357]/0.322) as compared to Model I. This analysis also showed female adolescents had more likely to report poor self-rated health compared to male counterparts (β = 0. 20, *p* = 0.018). However, this model revealed poor SRH was negatively associated with elementary educational status of adolescents (β = −0.38, *p* < 0.001), secondary educational status (β = −0.47, *p* < 0.001), living in rural areas (β = −0.24, *p* = 0.048) and religiosity (β = −0.16, *p* < 0.001). In other words, the more adolescents involved in religious activities, lived in rural area and attended schools, the less they had experienced poor SRH status **(**Table 3 **).** Other social determinants such as wealth status, parental education and age were not significantly associated with SRH (*P* > 0.05). Combined, model 2 accounted for only 8.4% of random intercept variance estimates change on SRH ([1.038–0.951]/1.038). Model III adds other multiple risk factors (lifestyle such as drinking alcohol, smoking cigarettes, chewing khat), social support, and exposure to physical health, chronic household food insecurity, dietary diversity, BMI and presence of someone who smokes in the household. Accordingly, adolescents living with someone smoking in the household (β =0.27; *p* < 0.024), those who were exposed to multiple risk factors (e.g. chewing khat, smoking cigarettes, drinking alcohol) (β =. 11, *p* < 0.022), those who had physical health (illness) problems in the last one month (β = 0.52, *p* < 0.001) and female (β =. 0.19, *p* = 0.023) had increased risk of poor self-rated health status. However, this model depicted adolescent education (primary β = −0.38, *p* = 0.001), secondary (β = −0.47, *p* < 0.001); religious practice (β = −0.17, *p* < 0.001), rural residence (β = −0.31, 0.016) were significantly associated with better self-rated health status. After adjusting for covariates, this model revealed the effect of food insecurity persists (β = 0.28, *p* < 0.001) but the effect of food insecurity on health status decline from 0.328 to 0.281 by 16.7% compared to the previous model .The random intercept variations in the SRH predicted by this model declined by 8.9%(0.951–0.866)/0.951) compared with the second model and the entire final model accounted for 16.6% of variances compared to model I. The full model also showed a decrease in the effect of food insecurity on self-rated health status by 27.0%(0.281–0.357]/0.281). More importantly, the final model showed self-rated health is more pronounced and had linear trend for some time (β =2.11, *P* < 0.001) and started to decline later (β = −0.38, *p* < 0.001) regardless of their food security status (Tables 3 & 4 **)**.

**Table 3:** Random intercept coefficients estimated with maximum likelihood, Jimma Longitudinal Family Survey of Youth, Southwest Ethiopia (*n* = 1919)

	Model I		Model II		Model III	
Exposure to FI	β	SE	β	SE	β	SE
Food insecurity	0.357**	0.079	0.322**	0.081	0.281**	0.082
Time	1.850**	0.291	1.966**	0.308	2.109**	0.312
Time^2^	−0.327**	0.071	−0.356**	0.079	−0.381**	0.080
Socio Economic Positions						
Sex (female)			0.195*	0.082	0.189*	0.083
Age_(14–17)_			0.082	0.105	0.084	0.117
Age_(18–22)_			0.214	0.175	0.166	0.191
Education-Primary			−0.380**	0.113	−0.377**	0.113
Education-Secondary & above			−0.467**	0.131	−0.467**	0.131
Female headed household			0.015	0.111	0.039	0.119
Parental education						
Primary			−0.063	0.116	−0.065	0.114
Secondary & above			−0.091	0.117	−0.121	0.117
Wealth status			−0.030	0.059	−0.064	0.063
Rural residence			−0.242*	0.122	−0.308*	0.127
Religiosity index			−0.164**	0.042	−0.168**	0.041
Risk factors and Social support						
Multiple risk factors					0.114*	0.050
Smokers in the household					0.268*	0.119
Chronic HH food insecure					−0.109	0.088
Dietary diversity score					−0.054	0.041
Had social network & Support					0.061	0.043
Underweight					−0.035	0.084
Overweight					−0.565	0.328
Physical health (illness) in the last one month					0.521**	0.091
_Cons	−3.336**	0.087	−3.114**	0.299	−3.343**	0.316
Variance (_Cons)	1.038	0.155	0.951	0.141	0.866	0.137
ICC	23.99	0.027	22.42	0.026	20.84	0.026

*FI* Food Insecurity, *SE* Standard Error, *HH* Households, *Cons* Constant, *ICC* Intra Cluster Correlation

*P < =0.05, **P < 0.001;

**Table 4:** Results of fixed effects models, Jimma Longitudinal Family Survey of Youth, Southwest Ethiopia

Fixed effects estimates	Model I		Model II		Model III	
Exposure to FI	B	SE	B	SE	B	SE
Food insecurity	0.447**	0.105	0.454**	0.105	0.444**	0.106
Time	1.713**	0.304	1.954**	0.332	2.045**	0.338
Time^2^	−0.296**	0.075	−0.391**	0.092	−0.408**	0.094
Socio Economic positions						
Age_(14–17)_			0.161	0.174	0.166	0.175
Age_(18–22)_			0.484	0.326	0.441	0.329
Education-Primary			−0.096	0.181	−0.091	0.182
Education-Secondary & above			0.052	0.251	−0.050	0.253
Other Risk factors						
Multiple risk factors					0.079	0.066
Smokers in the household					0.066	0.166
Underweight					−0.076	0.123
Overweight					−0.490	0.426
Had illness in the last 1 month					0.273*	0.110

*FI* Food Insecurity, *SE* Standard Error

*P < =0.05, ** *P < 0.001*

**Fig. 2:**
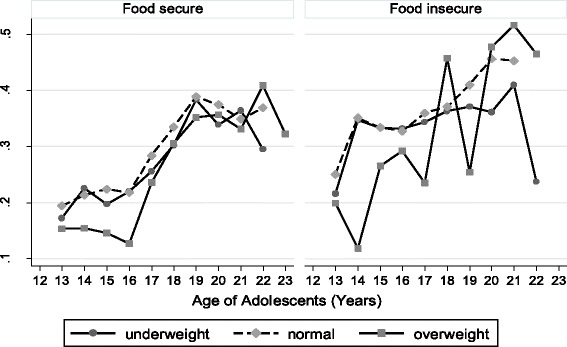
Predicted values of self-rated health by BMI, age and food security status, Jimma Longitudinal Family Survey of Youth, Southwest Ethiopia

Figure 2 shows predicted values of self-rated health by BMI, age and their food security status. Among food insecure group, the predicted value for SRH was less stable as the age of adolescents increases from 13 to age 22 for underweight, overweight and normal individuals. To be specific, as the age of adolescent increases from 13 to 22, slight drop-off predicted SRH was observed for underweight but large variations (instability) in predicted SRH was observed among overweighed one. For normal individuals, however, predicted SRH steadily increasing as compared to overweight and underweight individuals (Fig 2).

### Interaction term

Table 5 reports the interaction effect of food insecurity and time. Accordingly, the main effect of food insecurity on SRH has increased from 0.28 to 0.85 by 67.2% suggesting the persistent effect of food insecurity on self-rated health and also poorer SRH was increased with time (β =0.71,*p* < 0.001). More importantly, the interaction terms showed food insecurity had lower effect (β = −0.23, *p* = 0.027) compared to the previous model without interaction highlights there are other determinants responsible for the better SRH (Table 5).

**Table 5:** Random and fixed estimates for interaction effects of food insecurity with time on the adolescents self-rated health status, Jimma Longitudinal Family Survey of Youth, Southwest Ethiopia

Variables	Random intercepts estimates		Fixed effect estimates	
Random effects estimates	β	SE	B	SE
Food insecurity	0.846**	0.224	1.035**	0.272
Time	0.712**	0.068	0.682**	0.095
Food insecurity*time	−0.228*	0.103	−0.235*	0.126
Age_14–17_	0.065	0.104	−0.084	0.163
Age_18–22_	−0.090	0.166	−0.276	0.283
Education-Elementary	−0.366**	0.113	−0.123	0.180
Education-Secondary and above	−0.483**	0.130	−0.082	0.249
Risk factors	0.104*	0.049	0.065	0.066
Someone smoke in the Household	0.334**	0.117	0.155	0.164
Underweight	−0.034	0.084	−0.069	0.122
Overweight	−0.588	0.328	−0.509	0.422
Physical health (Illness)	0.466**	0.089	0.222*	0.110
Head of the HH (Female)	0.043	0.111		
Gender (female)	0.178*	0.083		
Parent Education (Primary)	−0.076	0.115		
Parent education (Secondary & above)	−0.123	0.116		
Wealth index	−0.044	0.063		
Rural	−0.156*	0.041		
Religiosity index	−0.154**	0.042		
Chronic food insecure HH	−0.127	0.089		
Diet diversity	−0.046	0.041		
Social support	0.060	0.043		
_Cons	−2.254	0.225		
Variance (_Cons)	0.859	0.136		
ICC	20.7	0.026		

*FI* Food Insecurity, *SE* Standard Error, *HH* Households, *Cons* Constant, *ICC* Intra Cluster Correlation

*P < =0.05, **P < 0.001

## Discussion

Examining exposures to risks and vulnerabilities from life course perspectives contributes to the existing knowledge in understanding the pathways by which the social determinants of adolescent health such as food insecurity impacts current adolescence and later adult life.

Despite the plausible biological mechanisms of food insecurity to cause poorer health outcomes, the longitudinal relationship has not been documented in low and middle income countries in general and Ethiopia in particular. So exploring such evidence is also of paramount importance in designing proper social and public health interventions at an early stage.

We found out that significant proportion of adolescents were food insecure overtime and the effect of food insecurity on poor self-rated health was observed among adolescents living in south west Ethiopia indicating food insecure adolescents were suffering poorer SRH and the effect of food insecurity on SRH also persists overtime. In addition, the differentials association of food insecurity on SRH also changed over time. Interestingly, the trends of poor self-rated health was not also constant across adolescence period where our quadratic term revealed it was more pronounced for at their middle adolescence and started to decline later. Furthermore, this study depicted the interaction effect of time with food insecurity influencing SRH status i.e. food secure adolescents could also rate poorer SRH. Prior research had also documented the association of food insecurity and poorer health status with few exceptions focus on its effect on the longitudinal development of SRH over time [5, 26, 28, 47–50].

As we stated above, this age is a critical period where the exposure to such events might mediate the lasting impact on later life, causing far reaching consequences not only on the general health but also on their mental, psychological and social wellbeing.

In this study, the magnitude of SRH was high and increased after some time of follow up. However, it looks stable between period 2 and period 3 (see Fig 1). Similarly, the coefficients of linear and quadratic terms depict that there was a rise in trend followed by a decline in poorer SRH. That means there are differentials and un-stability during early and later period of adolescence in complaining poor SRH. Similar studies showed SRH status among younger adolescents appears to be more characterized as enduring self-concepts and as the age increases SRH assume a spontaneous self-concept [35]. The reason could be due to their socio psychological process related to healthy development or maybe as they move from younger to late adolescence the shift of self-assessment might occur from enduring to spontaneous self-concepts. Therefore, this finding highlights the need to investigate differential characteristics of adolescents ‘self-health assessment. Although adolescents usually reported low perceived susceptibilities and their experiences, the magnitude of SRH across each round of the survey was very high in respective of their food security status. Similar studies depicted the magnitude of SRH ranged from 10 to 25% [51, 52].

There are several hypotheses to explain the differences in reported SRH during this specific age group. First, the constructs involved in understanding SRH during adolescence period might be one of the hypotheses. Studies have shown the measure of SRH is a complex and multi- dimensional judgment of individuals overall wellbeing could reflect domains beyond their physical health involving psychological wellbeing, behaviors and lifestyle factors. Self-rated health in particular was predicted by an overall sense of functioning combining of personal, socio-environmental, behavioral, psychological, and physical health factors [23, 35, 51, 52]. In other words, the health status of adolescent during the time of data collection might affect their self-rating. The reference framework used while rating their SRH status was not clear in the present study (e.g. they might refer the stock of physical, functional, socio psychological or tap relatively proximal factors determining their perceptions). As there is limited information on SRH among adolescents in Ethiopia, it remains difficult for us to understand the domains that compose the SRH constructs. Second, the formation of cognitive concepts of health during this age group (since adolescence is characterized by physical and psychological changes from early to late adolescence period) might affect their rating status. Thus, the health status perceptions and its determinants might be expected to change across their age. Third, we expect the difference in methods in terms of how the SRH was measured, categorized, the questionnaire is completed (i.e., self-completed or via face-to-face interview) and aspects of study design might also explain the difference in SRH across different studies [53].

This study showed adolescents who practiced multiple risk behaviors such as drinking alcohol, using khat; those who lived with smokers in the household and had history of recent illness were significantly associated with poor SRH. Similar studies indicated health-related behaviors such as smoking, alcohol consumptions and etc. could be associated to poorer health [54]. Nonetheless, educated adolescents had better SRH compared to those who never went to school. This can be attributed to a number of factors. For example, education is helpful to have better knowledge of health and better copping abilities. In addition, religiosity tends to be favorably associated with better SRH. Consistently, other studies also showed that individuals who are engaged in religious pursuits tends to have higher subjective wellbeing [55]. This highlights the role of religiosity in buffering the effects of stressors or the psychosocial resource that the religion provides might explain [56]. Religious involvement is commonly mediated by lifestyle choice, coping behaviors and high social network. According to resilient theories, individuals who are vulnerable due to poor socio-economic resources, low social capital, low economic capital or poor social networks may show stronger associations between self-rated health and religiosity. But further research is recommended to assess its effect, mechanisms and the causal directions being religiosity affect the SRH of adolescents living in Ethiopia.

Assuming the relationship between objective and subjective health assessment might change across adolescence life transitions (from early, middle and late), age graded differentials in reporting poorer SRH were assessed and no statistically significant association was observed. However, the regression coefficients showed that late and middle adolescents reported worse SRH than early adolescents suggesting the spontaneous assessment might have accounted for the relationship between food insecurity and poorer health controlling for other covariates.

More importantly, in this study, there was differential in SRH by gender where female reported more likely poor SRH than male counterparts. This might be due to the difference in exposure to biological, behavioral, environmental and social determinants of health and illness. For example, studies hypothesize the presence of “gender bias” associated to exposures to risks and illness although female subjects have biological and behavioral advantages [20, 26, 57].

### Strength and limitations of the study

Longitudinal follow up and representative sampling of adolescents are the strength of the study. Epidemiologic studies providing information on SRH of adolescents at population level are relatively rare. Previous cross sectional analysis of the same data showed food insecurity was associated with poor self-rated health. But, the current longitudinal analysis further strengthen existing literature. This study tries to show by identifying SRH as either spontaneous health or as an enduring self-concept among adolescents living in poor income settings. The present studies also demonstrate the merit of using one item measurement to assess adolescent’s health. There are however, considerable limitations. First, the appropriateness of SRH measures for adolescent health is not validated and the dimension of health it captures is not clear. Furthermore, the exact wording we used in SRH question “in general, how would you rate your health, today?” might be sensitive to short time (as we specified) and also it is different from other surveys across many countries. Therefore, in anyway, our responses may be influenced by short term fluctuations of health that can add noise to our interpretations; it is difficult to directly compare across surveys or it is difficult to know whether SRH variations across countries are due to true health difference or due to the use of different measurement of SRH. Second, there might be differences among individuals in rating from objective experience and or subjective health perceptions. Not all respondents use the same frame of reference in rating their subjective health. The concepts of SRH by itself might be different across social class, age, perceived social status and gender. Third, although both food insecurity and self-rated health was measure using 4–5 measurement scale, we were opted to dichotomize for this analysis. This is due to the presence of many zeros (rightly skewed). Thus, the impact of food insecurity on SRH may be an artifact of this process. In addition, both food insecurity and SRH are based on self-reported conditions and we cannot rule out the possibility of reverse causality (i.e. poorer SRH might also lead to lower food insecurity). As indicated by Jylha et al., the response to the self-rated health question sometimes are more intuitive, sometimes more consciously reflective [54]. More importantly, we recommend further research using qualitative approaches to assess the cultural or cognitive process associated with assessment of their health; to know which factors they took into account and how these factors influence their health; analyze adolescents own explanations that leads them to conclude in general their health is “good’ or “poor”. We also further recommend objective measures of health in understanding the biological basis of adolescent self-rated health, its association with different biomarkers (e.g. allostatic loading) health than relying individual’s subjective rating. More importantly, the mechanism by which food insecurity leads to poorer SRH needs further investigations.

## Conclusions

These findings suggest the presence of trajectories of poor SRH in food insecure adolescents. It adds information on prior knowledge suggesting exposure to food insecurity is strongly associated with poorer SRH during adolescence. There was also differentials in reporting poor SRH by their age and gender i.e. self-rating of health during adolescents fluctuates by their age and females were more likely to report poor health outcomes. Hence, intervention aimed at promoting adolescent health and wellbeing should not only focus on behavioral and psychological modifications but also the broader social and economic structure contributing to the food insecurity. This will help to reduce the deleterious effect of food insecurity on the health status of adolescents and minimize its lasting impact during their adulthood. More importantly, the study showed other social determinants of health such as education, place of living, religiosity could also be associated with better SRH. However, the effect of others determinants need further explorations.

## Additional file

Additional file 1: Table S1. Supplementary table of collinearity diagnostics**.** This is a description of the collinearity of the final model as indicated by its condition number and variation inflation factor.

## Abbreviations

ANOVA: Analysis of variance
BMI: Body mass index
ICC: Intra cluster correlation
JLFSY: Jimma longitudinal family survey of youth
SD: Standard deviation
SRH: Self rated health
WHO: World health organization
