# Supplementary material for: The effect of food insecurity on health status of adolescents in Ethiopia: longitudinal study
Source: BMC Public Health. 2017 May 18;17:465. doi: 10.1186/s12889-017-4406-5 (PMC5437384; doi:10.1186/s12889-017-4406-5)
Supplement: Additional file 1: Table S1. — Supplementary table of collinearity diagnostics. This is a description of the collinearity of the final model as indicated by its condition number and variation inflation factor. [file 12889_2017_4406_MOESM1_ESM.docx]

**Table S1. Supplementary table of collinearity d**iagnostics

| Variable | VIF | SQRT of VIF | Tolerance |
| --- | --- | --- | --- |
| Adolescent Food insecurity | 1.11 | 1.05 | 0.8997 |
| Time | 1.98 | 1.41 | 0.5059 |
| Sex | 1.09 | 1.03 | 0.9194 |
| Age 14-17 | 2.01 | 1.42 | 0.4986 |
| Age 18-22 | 3.19 | 1.79 | 0.3130 |
| Education-Elementary | 1.68 | 1.30 | 0.5689 |
| Education-Sec &Above | 2.10 | 1.45 | 0.4771 |
| Female Headed HH | 1.13 | 1.07 | 0.8816 |
| Parental education-primary | 1.35 | 1.16 | 0.7423 |
| Parental education –Sec&above | 1.95 | 1.40 | 0.5131 |
| Wealth index | 2.42 | 1.56 | 0.4129 |
| Rural | 2.31 | 1.52 | 0.4331 |
| Religiously index | 1.10 | 1.05 | 0.9067 |
| Multiple risk factors | 1.07 | 1.05 | 0.9321 |
| Smoke | 1.05 | 1.02 | 0.9547 |
| Chronic HH food insecurity | 1.21 | 1.10 | 0.8269 |
| Diet diversity | 1.05 | 1.02 | 0.9543 |
| Social network and support | 1.21 | 1.10 | 0.8256 |
| BMI-Underweight | 1.11 | 1.05 | 0.9045 |
| BMI-Overweight | 1.02 | 1.01 | 0.9825 |
| Poor physical health (illness) | 1.05 | 1.03 | 0.9516 |

Note: VIF; Variance inflation factor; SQRT=Square root

Condition Number 14.6269

Eigenvalues & Cond Index computed from scaled raw sscp (w/ intercept) Det(correlation matrix) 0.0188

Conclusion: VIF and condition number fall within acceptable rule of thumb.
